# Conductivity of the phloem in mango (*Mangifera indica* L.)

**DOI:** 10.1038/s41438-021-00584-1

**Published:** 2021-07-01

**Authors:** Miguel Barceló-Anguiano, José I. Hormaza, Juan M. Losada

**Affiliations:** Instituto de Hortofruticultura Subtropical y Mediterránea La Mayora (IHSM La Mayora-CSIC-UMA), Avda Dr. Wienberg s/n. 29750, Algarrobo-Costa Málaga, Spain

**Keywords:** Plant physiology, Plant transporters

## Abstract

Mango (*Mangifera indica* L., Anacardiaceae), the fifth most consumed fruit worldwide, is one of the most important fruit crops in tropical regions, but its vascular anatomy is quite unexplored. Previous studies examined the xylem structure in the stems of mango, but the anatomy of the phloem has remained elusive, leaving the long-distance transport of photoassimilates understudied. We combined fluorescence and electron microscopy to evaluate the structure of the phloem tissue in the tapering branches of mango trees, and used this information to describe the hydraulic conductivity of its sieve tube elements following current models of fluid transport in trees. We revealed that the anatomy of the phloem changes from current year branches, where it was protected by pericyclic fibres, to older ones, where the lack of fibres was concomitant with laticiferous canals embedded in the phloem tissue. Callose was present in the sieve plates, but also in the walls of the phloem sieve cells, making them discernible from other phloem cells. A scaling geometry of the sieve tube elements—including the number of sieve areas and the pore size across tapering branches—resulted in an exponential conductivity towards the base of the tree. These evaluations in mango fit with previous measurements of the phloem architecture in the stems of forest trees, suggesting that, despite agronomic management, the phloem sieve cells scale with the tapering branches. The pipe model theory applied to the continuous tubing system of the phloem appears as a good approach to understand the hydraulic transport of photoassimilates in fruit trees.

## Introduction

The phloem is the vascular tissue that transports photoassimilates from the source photosynthetic organs towards the sink tissues, which are the growing organs of the plant, including the roots, the meristems, fruits, or the flowers and inflorescences^1,2^. This continuous piping system hydraulically drives substances at long and short distances, and works according to the osmotically generated pressure flow hypothesis, proposed almost a century ago^3^, but empirically tested in relatively few woody plants^4–6^. The hydraulic function of the phloem is intimately linked with the geometry of the individual sieve tube elements, which are connected in series composing a continuous tube and, thus, follows the laws of an electric circuit with resistances to the flow (the connections between tubes or sieve plates). The geometrical scaling of the conductive elements composing the phloem along the trunks of trees have remained unexplored until recently, when the development of microscopy techniques has allowed detailed visualisation of these micro conduits, most remarkably the quantification of the micro pores composing the sieve plates^7^. As a result, the geometrical scaling of the phloem sieve elements has been revealed in the stems of forest trees^4,6,8^, and in tapering branches of shrubs^5^. Scaling of the sieve tube elements correlates with the increasing size of the pores composing the sieve plates, which offer most of the resistance to sap flow in the phloem, as evaluated by the most recent models of hydraulic transport^9,10^. While growing evidence of this scaling geometry of the phloem, sieve tubes appears to characterise woody organisms, including the variability observed in lianas^11^, only a handful of studies have correlated phloem structure and function in fruit trees, despite the fact that the phloem plays a critical part on survival, fructification, and response to stress.

Mango (*Mangifera indica* L.) ranks fifth worldwide in production among fruit crops, after bananas, apples, grapes, and citrus, and its trade and production at the global scale has gradually increased in the past decade (according to FAO^12^). Mango is a woody perennial fruit tree crop belonging to the family Anacardiaceae, that displays monopodial growth, and both the leaves and the inflorescences develop on the youngest terminal branches^13^. Although mangoes are originated from tropical Asia, they can also be cultivated in regions with subtropical climates, and low temperatures (even light freezes can seriously damage the trees) are the main limiting factors to its cultivation at higher latitudes^14–19^. Compared with the big size of mango trees in tropical environments, low winter temperatures reduce their growth in subtropical climates, and, along with pruning, help to keep mango trees at a manageable height, resulting in small trees with compact tapering branches^20^. Recent computer simulations of mango tree architecture have shown that the allometric relationships between biomass production (i.e., leaves) and branch vigour are influenced by the hydraulics of branches, thus affecting productivity^21^. An example of this relationship was shown by measurements of the vascular tissues composing the stem cross-sectional areas, which revealed that higher phloem to xylem ratios correlated with less vigorous or dwarfing varieties^22^. Although counterintuitive, dwarfism is a desirable character in many tree crops, due to the easier agronomic management of smaller trees combined with good productivity. While some information on the hydraulic traits of the xylem tissue is available in mango, as in most woody perennial fruit crops, there are no reports to date on phloem structure in mango, even though this tissue is pivotal for tree productivity.

Mango trees are traditionally considered as drought-tolerant, and this has been correlated with anatomical adaptations, such as a taproot that reaches profound soil depths, or the massive presence of laticiferous canals, which putatively adjust the osmotic potential under drought stress^23^. Yet, there is a lack of detailed anatomical studies on the vascular tissues of this important fruit crop. The aim of this work is to provide novel information on the anatomy of the so far understudied sieve tube elements of the phloem in the stems of cultivated *M. indica*, and test whether sieve tube elements scale in the tapering branches of short sized fruit tree species. With this anatomical information, we modelled the conductivity of the phloem tissue, and compared these data with recent reports on phloem structure in other species. Altogether, these observations will set the base for future works on the effect of biotic and abiotic factors on long distance transport and, consequently, on the productivity of mango trees, leading to a better agronomic management of this and other tree crops.

## Results

### Anatomy of mango stems

**Fig. 1:**
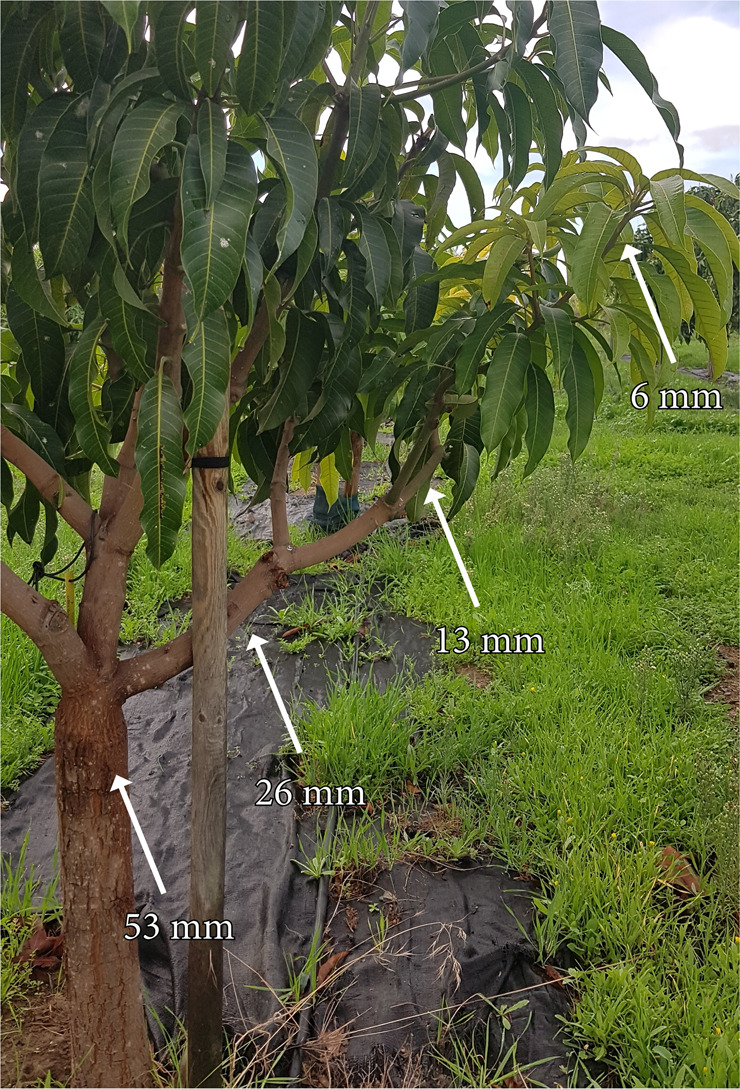
Six-year-old mango tree of the variety ‘Winters’ in the field. Arrows show the diameter of the tapering branches analysed in this study

**Fig. 2:**
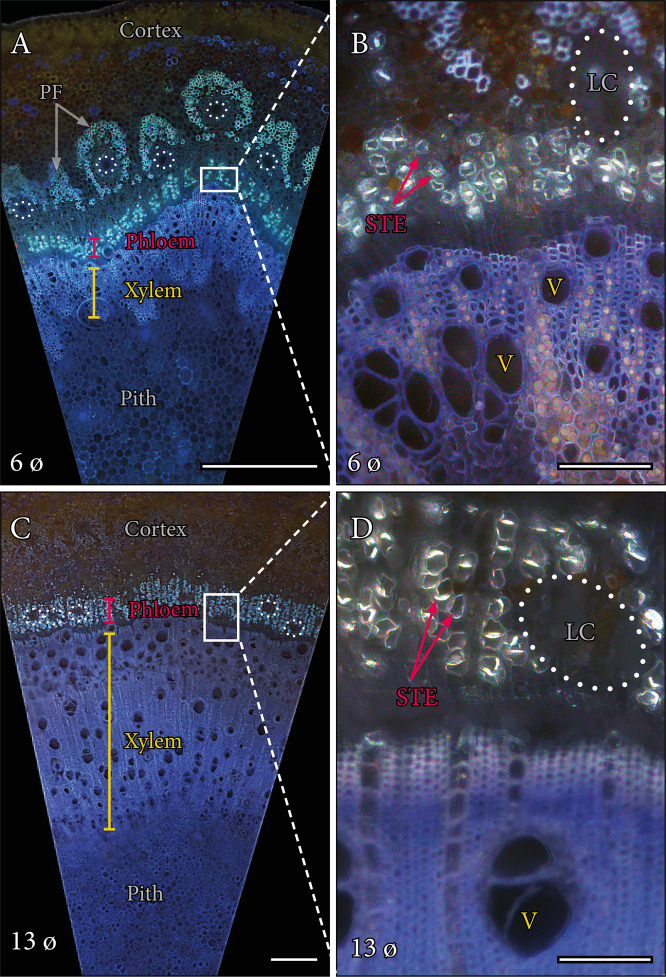
Anatomy of the stem in *Mangifera indica* ‘Winters’. **A** Cross sections of the 6 mm stem showing the different tissues from the most external (cortex), pericyclic fibres, through the phloem (red), the xylem (yellow), and the most internal pith. **B** Detail of the sieve tube elements of the phloem (red arrows) in cross section, showing the presence of callose in the lateral walls of the sieve tube elements. **C** Cross sections of a 13 mm diameter stem showing a larger proportion of the xylem tissue, and a fibre-less phloem with numerous laticiferous canals (dotted lines). **D** Detail of the phloem, showing the wider sieve tube elements with callose in their walls (red arrows), and the laticiferous canals embedded in the phloem tissue. Epifluorescence micrographs of cross sections of mango stems stained with aniline blue. LC laticiferous canals, PF pericyclic fibres, STE sieve tube elements, V vessels. **A**, **C** scale bars = 500 µm; **B**, **D** scale bars = 100 µm

Mango trees share a common body plan with branches that are mainly woody, except for the current year apical growth (6–8 mm width), which is typically soft (Fig. 1). Cross section of the current year flush displayed an extensive pith surrounded by a diffuse porous xylem; axial phloem areas around the xylem were interspersed with axial parenchyma; numerous laticiferous canals surrounded the phloem, and, at the most external side, pericyclic fibres protected them (Fig. 2A). Close up images of the phloem cross sections revealed the presence of callose not only in the sieve plate connections, but also in the lateral walls of the sieve tube elements, a feature that allowed their identification from the rest of the phloem cells (Fig. 2B). In branches of wider diameters, as the pith became smaller, the xylem constituted a wider part of the cross-sectional area, surrounded by a continuous phloem tissue devoid of pericyclic fibres (Fig. 2C). In those wider branches, the vascular elements of the xylem and the phloem showed wider radii, and the sieve tubes kept a callose-rich wall (Fig. 2D). Numerous laticiferous canals were interspersed with the sieve tubes of the phloem tissue.

### The phloem in the tapering branches of mango

**Fig. 3:**
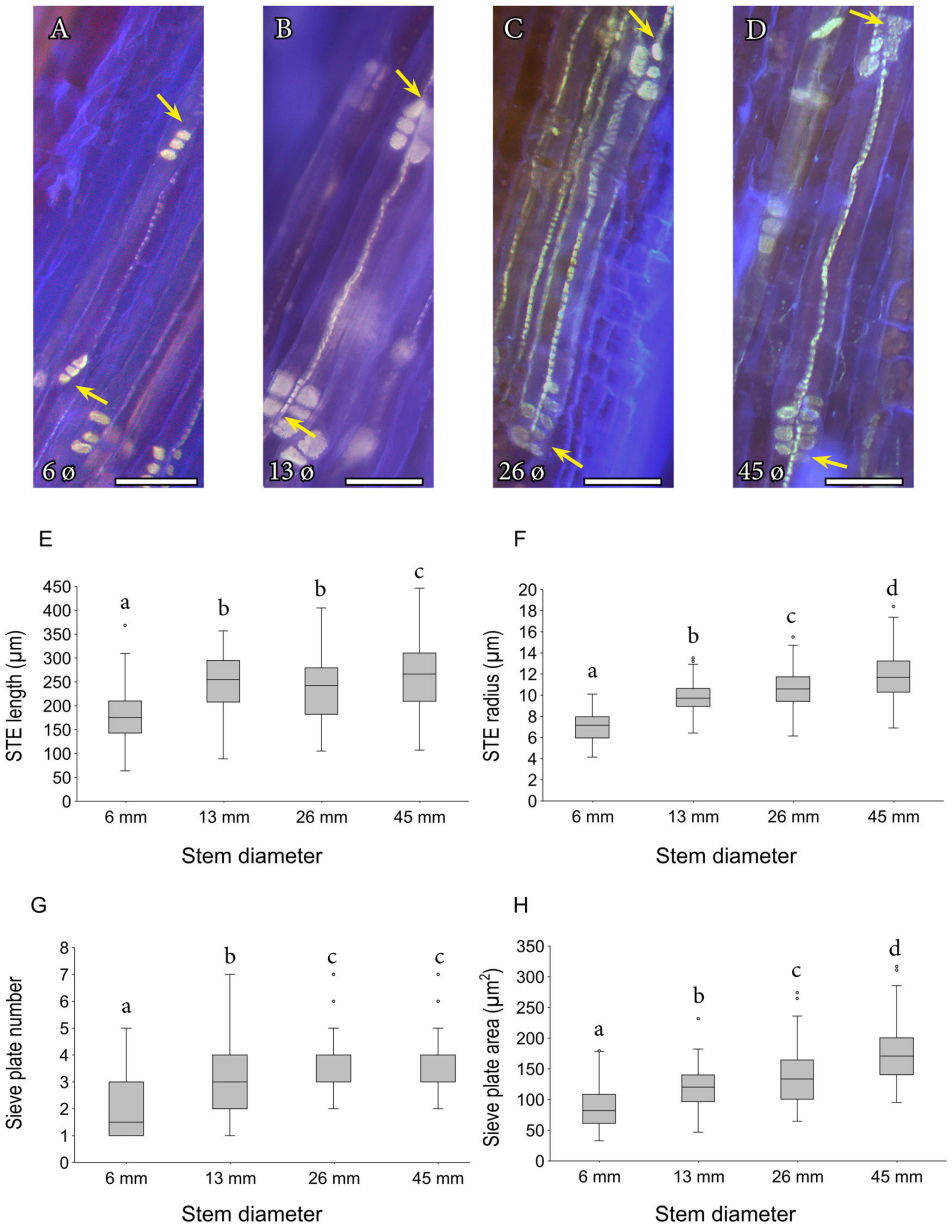
Morphology and geometry of the sieve tube elements in the stems of mango ‘Winters’. **A**–**D** Epifluorescence micrographs of sieve tube elements from branches of increasing diameters: 6 mm (**A**), 13 mm (**B**), 26 mm (**C**), and 45 mm (**D**). Yellow arrows show the compound sieve plate connections between the sieve tube elements stained with aniline blue (yellow colour), counterstained with calcofluor white for cellulose (blue colour). **E** Sieve tube element (STE) length at the four branch diameter ranges. **F** Sieve tube element (STE) radius at the four branch diameter ranges. **G** Number of sieve areas per plate connection between tubes at the four branch diameter ranges. **H** Area of each plate at the four branch diameter ranges. Letters over box plots indicate significant differences between branches (pooled data from both trees), following a one-way ANOVA and a Tukey test at *p* < 0.05. **A**–**D** scale bars = 50 µm

Longitudinal views of the sieve tube elements stained with aniline blue revealed that callose hyper accumulated in the sieve plates, whereas both callose and cellulose composed the sieve tube wall, as shown by counterstain with calcofluor white. The sieve tubes of mango typically associated in pairs, and sibling tubes shared numerous sieve areas in their lateral walls (Fig. 3A–D). The average sieve tube element length ranged from 174.6 ± 4.18 (SE) µm in the thinnest branches to 262.4 ± 5.99 (SE) µm in the thickest ones (Fig. 3E), following a logarithmic increase from current year branches to older ones (*y* = 59.1ln(*x*) + 179.9; *r*^2^ = 0.91). Similarly, the values of radii ranged from 7.04 ± 0.11 (SE) µm in the youngest branches to 11.8 ± 0.18 (SE) µm in the older ones, further increasing logarithmically (*y* = 3.37ln(*x*) + 7.14; *r*^2^ = 0.98; Fig. 3F). Strikingly, the number of sieve areas per compound sieve plate (*y* = 1.27ln(*x*) + 1.99; *r*^2^ = 0.97; Fig. 3G), as well as their individual size, scaled logarithmically with the diameter of the branch (*y* = 281ln(*x*) + 56.9; *r*^2^ = 0.97; Fig. 3H).

**Fig. 4:**
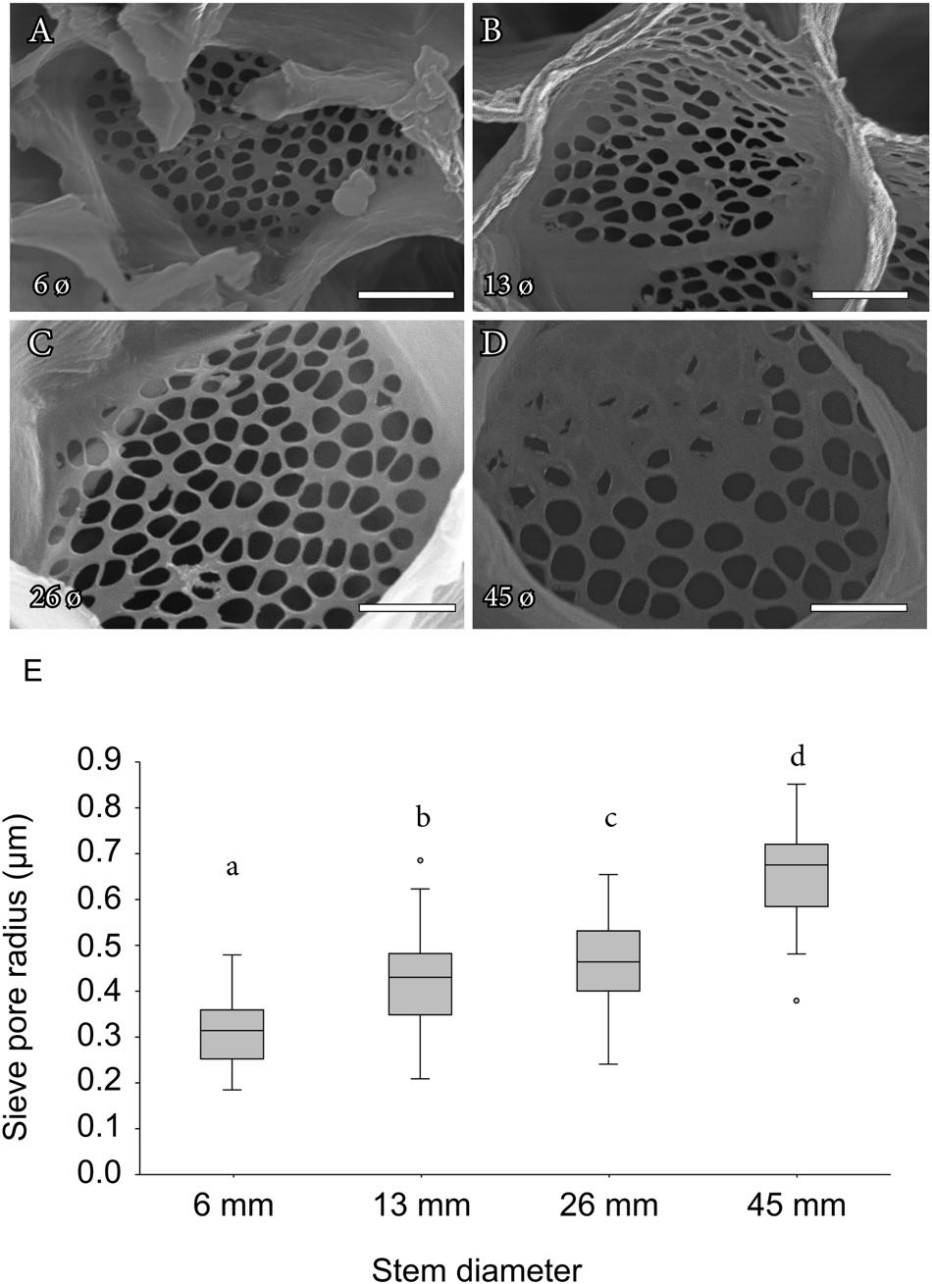
Sieve plate anatomy in mango stems. **A**–**D** Scanning electron microscopy images of the sieve plates of mango ‘Winters’ in tapering branches of 6 mm (**A**), 13 mm (**B**), 26 mm (**C**), and 45 mm (D). **E** Radius of individual pores in the sieve plates of each branch diameter (aggregated data from both trees). Letters over boxes display significant differences in pore size at each branch diameter following a one-way ANOVA and a Tukey test at *p* < 0.05. **A**–**D** scale bars = 4 µm

Scanning electron micrographs revealed that pore size increased gradually from the current year branches to older branches, following the transport pathway (Fig. 4A–D). Quantification of the total pore number per end tube, which included the number of sieve plates as a factor, displayed a logarithmic increase with branch diameter (*y* = 68.8ln(x)+185.4; *r*^2^ = 0.97; Supplementary Fig. 1), in line with the geometrical parameters of the tubes. The pore radius, on the other hand, further increased linearly with the diameter of the stem, roughly doubling its size from the thinnest [0.31 ± 0.01 (SE) µm] to the thickest [0.66 ± 0.01 (SE) µm] stems (*y* = 0.11*x* + 0.19; *r*^2^ = 0.93; Fig. 4E).

### Conductivity of the stems in mango

**Fig. 5:**
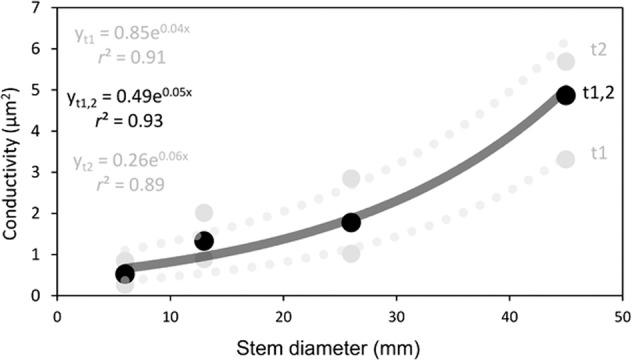
Conductivity (*K*) of the sieve tube of the phloem in the stems of *Mangifera indica*. The specific conductivity of the sieve tubes increased exponentially from the younger stems to the base of the trees, following an exponential function. *t*1, *t*2, trees 1 and 2, respectively, (grey circles and dotted lines); *t*1,2: conductivity of the sieve tubes after pooling data from both trees (black circles, grey line)

The specific conductivity of the sieve tubes in the stems of *M. indica* calculated by this method ranged roughly between 0.5 and 6 µm^2^ (Fig. 5). From the youngest green branches to the thickest stems, there was an increase in the conductivity of the tubes that fitted with an exponential function, even though the trees were managed at a height <2 m. Although the absolute values were different for the two trees, they both followed an exponential increase of the sieve tube conductivity.

## Discussion

### Callose as a marker of the sieve cells of the secondary phloem in mango stems

Although callose typically accumulates in the symplasmic connections between the sieve tube elements or sieve plates, we hereby revealed that the sieve tubes of the stems in mango trees contain abundant callose along their lateral walls, making them discernible from other phloem cells in cross sections. Elucidating the walls of the sieve tube elements of the phloem is challenging in most species, yet essential to understand the general conductance of the phloem tissue and, thus, the transport of photoassimilates at the whole tree level. Recently, monoclonal antibodies that attach to a branched pectin epitope present in the sieve tube elements of *Beta vulgaris* leaves were used as markers of the sieve tube walls^24^. More recently, these antibodies were used to label sieve tubes of members of the genus *Populus*^25^, pointing to a great potential of these markers for a general use in a wide range of plant species. The technique is relatively tedious, and requires physical (i.e., using liquid nitrogen) or chemical fixation of the tissue prior to the immunolocalization, which normally leads to turgidity loss of the sieve tube elements. In order to avoid the fixation step, our work with mango showed that staining fresh sections for callose with aniline blue can be an inexpensive, fast, and relatively easy tool to identify the sieve tubes. The use of aniline blue to measure sieve tube element length in longitudinal fresh sections has been used for decades^4–6^, but callose presence in the sieve tube walls did not show consistency across the species evaluated. Some examples that previously detected callose on the sieve tubes of stems include the trees *Bombax buonopozense* and *Sterculia tragacantha*^26^ or the shrub *Illicium parviflorum*^5^, where the pitted walls of the sieve tubes accumulated callose, and this was instrumental for quantifying the cross-sectional variation of the phloem tissue at different axial positions along the stem. The technique has some inconveniences, such as the difficulty in obtaining good quality fresh sections of the phloem in a short time, which is critical to maintain the integrity of the sieve tubes, or the stiffness of the stem, that can smash the flexible tubes against the rigid xylem. Despite all these inconveniences, and given the lack of evaluations of the phloem in fresh sections, we suggest that using aniline blue might be a good first approach of general use to understand the structure of the phloem in tree branches.

### Laticiferous canals of the phloem tissue

The presence of callose along the sieve tube element walls might be unique to the phloem of mango, but the question remains on the reasons behind the presence of this water insoluble polysaccharide, which typically acts as a sealing compound isolating cells after damage^27^, in the lateral walls of the phloem sieve tube elements. We can only speculate on the role of callose in the sieve tube walls, but it is noteworthy the presence of numerous laticiferous canals interspersed in the phloem tissue of mango. Laticiferous canals pervade the majority of vegetative and reproductive tissues in most species of the family Anacardiaceae^28^, and their role has been typically associated with defence against biotic predators, given the composition of the mucilaginous material that fills them, such as tannins, lipids, and polysaccharides^29^. From an anatomical perspective, the laticiferous canals associate with the phloem tissue, displaying positive turgor pressure due to the high concentration of osmolites putatively donated from the phloem^23^. The physiological role of laticiferous canals was previously studied in *Musa* sp.^30^, *Hevea brasiliensis*^31^, and *M. indica*^32^, and they seem to actively participate in the osmotic adjustment during periods of water scarcity. Callose of the sieve tubes may isolate the sieve tubes for the hydraulic function, but also provide with the mechanical resistance required to stand with positive pressures of the sap, especially during periods of drought^33^.

### Conductivity of the sieve tubes in the stems of mango

Previous models evaluating the effect of stem diameter in current year branches of mango trees revealed that they may be useful to predict leaf and branch conductivity^20,34^, which refers to the conductivity of the xylem, given that the xylem constitutes most of the branch mass. In addition, the geometry of the xylem vessels has been widely studied from the perspective of the scaling relationships between vessel size and branch width/height in forest trees^35–37^. Conversely, the anatomy of the sieve tubes has remained understudied in many woody species, such as *M. indica*. While current models likely overestimate conductivity—they assume an ideal scenario with empty sieve tubes, ignoring the presence of organelles and proteins^9,10^, they illustrate the influence of anatomical variation on hydraulics. We reported that, in mango trees, the geometry of the individual sieve tube elements followed a direct logarithmic relationship with branch diameter, but the calculated conductivity was exponential, sharply increasing from the current year growth towards the base of the tree. This is because the structure of sieve plate connections—size and number of pores—mainly influence conductivity or how easy sap flows between tubes. Furthermore, our evaluations revealed that the sieve tube elements contained compound sieve plates (more than one sieve area per end tube), along the branches of different widths. Compound sieve plates appear to dominate the stems of woody species from both tropical and temperate climates^4,8^, with some exceptions that account for ~20% of tree species^8^. In mango (and likely in many other woody species), the number and size of the sieve areas connecting the tubes scale with the branch order/diameter, suggesting that their anatomical features are determined according to the axial position of the vascular cambium. In fact, this scaling has been recently evaluated in the stems of *Quercus rubra* trees of different ages, which displayed compound plates^6^. The number of sieve areas at the base of the trunk in >5 m tall trees of *Quercus* tripled those of mango, in line with their longer and wider sieve tube elements, suggesting that the age of the trunk influences the general geometry of the sieve tubes, thus facilitating transport towards the base of the tree^6^. The mango trees studied in this work were ~2 m tall, and the geometry of the sieve tube elements falls within the values reported for *Quercus* trees of comparable heights^6^.

Fruit trees constitute an excellent system in which to explore the effect of age on the sieve tubes, since most varieties grafted onto rootstocks maintain the genetic age of the source tree. Our sampled trees were grafted 6 and 8 years ago, respectively, and, despite differences in some of the branches evaluated, sieve tube anatomy displayed a scaling geometry across branches of increasing vigour. This reinforces the idea that the allometry of the vascular conduits strongly correlates with branch robustness and, thus, the hydraulic conductivity of the phloem increases in a similar fashion to that of the xylem^21^. From a xylocentric perspective, both young^38^ and old^36,37^ forest trees showed similar scaling relationships. Our quantifications of the scaling relationship between sieve pore size and diameter of the stem further reflect a conserved trend across trees of the same genotype (the same variety), but of different ages^4,8,39^. We interpret this as another evidence that wider stems optimise the hydraulic conductivity of the phloem. As comparison, herbaceous vines of ~7 m long, which develop compound sieve plates towards the base of the stem, displayed similar pore sizes^40^, but resulted in lower conductivities than mango trees of 2 m height. The higher number of sieve areas per end tube in woody species might then constitute a key facilitator of the hydraulic conductivity of the phloem.

## Concluding remarks

The unifying model that explains the structural and hydraulic function of trees, known as the pipe model theory^41^, has typically been applied to the xylem, whose scaling depends on the environment, plant height, and other important internal and external features of the plant^37^. In contrast, factors affecting phloem architecture are starting to be unravelled in trees, and suggest similar scaling relationships to those found in the xylem, but they deserve a careful examination in both forest and crop trees.

## Materials and methods

### Plant material

We used two adult clones of the mango cultivar ‘Winters’, grafted onto polyembrionic Gomera-4 rootstock, 6- and 8-year old, respectively, both belonging to the germplasm collection of the Institute for Mediterranean and Subtropical Horticulture ‘La Mayora’ in Málaga, south of Spain (X: 407.162,62; Y:4.068.652,56; UTM:30). Given that the trees are pruned to keep a manageable canopy, we sampled branches of increasing diameters, from the current year branches to the base of the trees, following the protocol previously used for shrubs^5^. Thus, we selected three branches per tree oriented to all directions, and, in each branch, four diameters with increasing thickness: between 6–8 mm, 13–15 mm, 26–28 mm, and 44–46 mm (Fig.1).

### Fluorescence microscopy: sieve tube geometry

Branch portions per sample point that contained the phloem tissue (i.e., the external part between the xylem and the bark), were collected with a sharp knife, and placed in 1× PBS (phosphate-buffered saline), for transportation to the laboratory. Then, transverse (i.e., perpendicular to the branch length) and longitudinal (i.e., parallel to the xylem tissue) hand sections of ~1 mm thickness were obtained with a microscalpel, mounted on slides, and stained. The samples were stained either with just aniline blue (0.1% w/v in 0.1% K_3_PO_4_)^27^, which labels the polysaccharide callose, typically accumulated in connections between sieve tubes of the phloem—the sieve plates—or with aniline blue counterstained with 0.1% w/v calcofluor white in 10 mM CHES buffer with 100 mM KCl (pH 10)^42^, which stains the cellulose of the cell walls. These sections were observed with a LEICA DM LB2 epifluorescence microscope (Leica Mycrosystems, Wetzlar, Germany), using a 405 nm filter barrier, equipped with a Leica DCF310 FX camera and a LAS V4.5 software.

### Scanning electron microscopy: sieve plate pore size and density

Small portions of the branches from the same areas sampled above were cut and immediately submerged in liquid nitrogen to avoid hyper accumulation of callose in the sieve pores as a response to damage, and transferred to super chilled ethanol. They were then incubated overnight in Falcon tubes at −80 °C, and gradually transferred to increasing temperatures of −20 °C for 24 h, −4 °C for 24 h, and room temperature. The samples were then sectioned in ethanol at 1–2 mm using a double-edged razor blade. These sections were washed thrice in distilled water during 2–3 h to remove the ethanol, and then incubated in a mixture of 0.1% w/v proteinase K dissolved in 50 mM Tris-HCl buffer, 1.5 mM Ca^2+^ acetate, and 8% Triton X-100, pH 8.0 in a water bath at 60 °C for at least 14 days, replacing the mixture every week^7^. Once the cytoplasmic content was digested, sections were rinsed once in ethanol, and then twice in deionized water. These sections were incubated again in a water bath at 60 °C for two days in 0.1% alpha amylase, which removes the starch accumulated in the sieve plates. After that, they were rinsed again in water to remove debris, and the water was poured prior to lyophilisation with a freeze-drier (CoolSafe 4–15 L Freeze Dryers, LaboGene, Allerod, Denmark) for 24 h. Desiccated samples were mounted on SEM studs, covered with gold-palladium using a sputter coater (QUORUM Q 150 R ES), and finally observed using a scanning electron microscopy (JEOL JSM-840).

### Image and data analysis

Using the fluorescence microphotographs, 75 tubes per sampling point and tree were evaluated to determine tube length and radius, resulting in a total of 600 tubes analysed. A total of 50 tubes were further measured per sampling point, and tree to determine sieve plate number and individual sieve plate area (*n* = 400). The number of sieve pores evaluated was different between trees, because observation of the sieve plates with scanning electron microscopy depends on their exposure on the surface of sections, which is rarely consistent across samples. Thus, we pooled the data from both trees to make 75 pores measured per sampling location. From these samples, we measured pore radius, pore density, evaluated as the number of pores in a defined area of the images, and the total estimated number of pores (number of pores per area multiplied by the area of the sieve areas and by the number of sieve areas). All images were analysed with the ImageJ-Fiji software^43^.

Statistics were performed using SPSS 23.0 (SPSS Inc., Chicago, USA). All data followed a normal distribution, except pore size, and density, which were log-transformed to fit normality. We found differences between trees in some of the parameters evaluated, but they were not consistent across sampling positions, and this could be attributed to the different vigour of each tree^6^. Given that our goal was to evaluate sieve tube escalation across branches, we aggregated data to perform a one-way ANOVA test, which displayed differences between geometrical parameters.

### Conductivity of the phloem along the stems of mango

With the anatomical data obtained, we computed the conductivity of the phloem in the stems of mango, following the Hagen–Poiseuille model of laminar flow through cylinder pipes:

1 $$U = k{\Delta}p/\eta L$$

where *U* is the velocity of the flux, Δ*p* the differential pressure between the source and the sinks, *η* the viscosity of the fluid, and *L* the length of the tube. The first three parameters require in vivo measurements, and so far there are no methods available to get measurements of fluid viscosity, pressure, or velocity in woody stems, with the exception of radioisotopes used to measure velocity^44^. Thus, we focused on the evaluation of the conductivity *k*, which is greatly influenced by the structural variation of the sieve tube elements along the plant as previously reported^40^. For the calculation of conductivity, we used the mathematical model developed by Jensen et al.^9,10^, that has been applied to different systems, such as long vines^40^, tall trees^4,6^, or shrubs^5^. This method establishes that the hydraulic resistance of a sieve tube is composed by the sum of two factors, the resistance of the lumen (the axial wall of the tube) plus the resistance of the sieve plate (the connection between tubes), as to:

2 $$R = R_{\mathrm{L}} + R_{\mathrm{P}}$$

The lumen, assumed in this work as a cylinder without lateral leakage, offers a resistance that is directly proportional to the viscosity (taken from standards^9,10^), and the length of the tube, and inversely related with the fourth power of the tube radius, as to:

3 $$R_{\mathrm{L}} = 8\eta l/\left( {\pi r^4} \right)$$

On the other hand, the sieve plate resistance is defined by the equation:

4 $$R_{\mathrm{P}} = \left( {3\eta 1/r_p^3N} \right)\left( {1/I\left( {\alpha ,\beta } \right)} \right.$$

where *N* is the total number of pores per end tube, and *I* is a function of two non-dimensional parameters *α* and *β* (ref. ^40^).

Finally, the conductivity (*k*), which is the inverse of the hydraulic resistance, follows the general equation^40^:

5 $$k = \eta L/\left( {\pi r^2R} \right)$$

## Supplementary information

Supplemental Figure 1
