# Supplementary figures and images for: Conductivity of the phloem in mango (Mangifera indica L.)
Source: Hortic Res. 2021 Jul 1;8:150. doi: 10.1038/s41438-021-00584-1 (PMC8245510; doi:10.1038/s41438-021-00584-1)

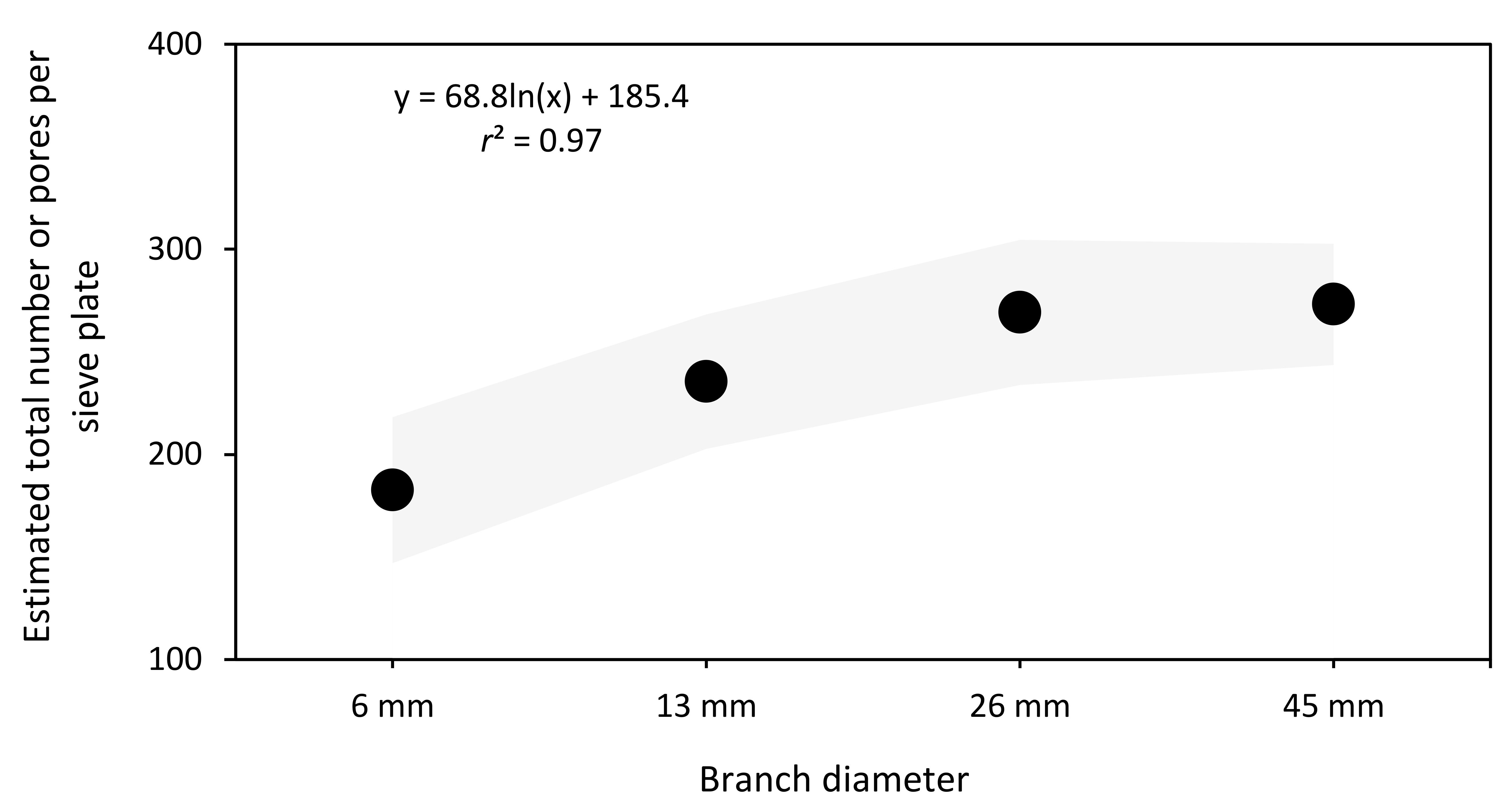

Supplement: Supplementary file 1 — Supplemental Figure 1 [file 41438_2021_584_MOESM1_ESM.jpg]
